# A Word of Caution: Aorto-Right Ventricular Fistula, an Uncommon Pitfall of Perceval Sutureless Valve

**DOI:** 10.3390/jcdd13060230

**Published:** 2026-05-28

**Authors:** Ziyad Gunga, Augustin Rigollot, Elsa Hoti, Zied Ltaief, Gabriel Saiydoun, Anna Nowacka, Valentina Rancati, Florine Valliet, Matthias Kirsch

**Affiliations:** 1 Department of Cardiovascular Surgery, Lausanne University Hospital (CHUV), 1011 Lausanne, Switzerland; 2 Department of Anesthesiology and Intensive Care, Lausanne University Hospital (CHUV), 1011 Lausanne, Switzerland; 3 Department of Cardiac and Thoracic Surgery, Pitié-Salpêtrière University Hospital, Assistance Publique des Hôpitaux de Paris, 75013 Paris, France

**Keywords:** aorto-right ventricular fistula, septal tear, perceval, SAVR, sutureless valve, pitfalls

## Abstract

Background: An aorto-right ventricular fistula (ARVF) secondary to membranous septum rupture is an exceptionally rare complication after surgical aortic valve replacement (SAVR). While sutureless prostheses such as the Perceval valve have gained wide acceptance due to reduced cross-clamp times and procedural simplification, the reported adverse events predominantly include conduction disturbances and paravalvular leaks. Structural septal disruption remains sparsely described. We report a case of an early ARVF after Perceval implantation and review the pathophysiological and procedural mechanisms implicated in septal injury following sutureless and transcatheter aortic valve interventions. Case Description: A 66-year-old woman with severe bicuspid aortic valve stenosis underwent SAVR via a median sternotomy using a Perceval XL prosthesis after meticulous annular decalcification and sizing. Immediate intraoperative transesophageal echocardiography (TEE) confirmed optimal seating without any paravalvular regurgitation. Within 24 h, the patient developed a complete atrioventricular block followed by cardiogenic shock. A repeat TEE revealed a large ARVF with significant left-to-right shunt. Emergent re-exploration identified a membranous septum tear. The Perceval prosthesis was explanted, the defect was closed with a reinforced patch repair, and a 27 mm Inspiris Resilia bioprosthesis was implanted. Peripheral veno-arterial ECMO support was required temporarily. The patient recovered and remained free of prosthetic dysfunction at the two-year follow-up. Discussion: Membranous septum rupture after AVR has an estimated incidence of 0.4–1.5% in TAVR cohorts but is virtually unreported with Perceval valves. The mechanisms are thought to be chronic radial stress from oversized or malpositioned prostheses. Case reports with TAVR devices emphasize oversizing as a risk factor. Predictive factors for septal injury in sutureless AVR mirror those for conduction disturbances: valve oversizing, shallow infra-annular septal length, heavy calcification, and prior valve surgery. Preventive measures, such as strict sizing protocols, the avoidance of balloon dilation, and optimized implantation depth, have reduced conduction complications and may mitigate septal trauma. The treatment choice, whether percutaneous or surgical closure, depends on hemodynamic stability, defect size and anatomy, and operative risk. Conclusions: Early ARVF after Perceval implantation is exceedingly rare but potentially catastrophic. Strict adherence to sizing principles, awareness of septal anatomy, and prompt management, percutaneous in selected stable cases or surgical in acute large defects, are essential to optimize outcomes in sutureless AVR.

## 1. Introduction

An aorto-right ventricular fistula (ARVF) secondary to membranous septum rupture represents one of the most dramatic and least anticipated complications following aortic valve replacement (AVR). While sutureless prostheses such as the Perceval valve (Corcym, Milano, Italy) have become popular for their reduced cross-clamp times and simplified implantation technique, their expanding use has been accompanied by a spectrum of device-related sequelae: most notably conduction disturbances and paravalvular leaks [1,2]. In contrast, the mechanical disruption of the membranous septum with a consequent septal defect and fistulization into the right ventricle remains exceptionally rare and sparsely described in the literature, particularly in the context of Perceval implantation. We present the case of a patient who developed a large ARVF within 24 h of Perceval valve implantation, requiring emergent explantation, septum repair, and valve re-replacement. Through a concise review of the literature on septal injury across both TAVR and sutureless AVR platforms, we explore the anatomical and procedural risk factors that predispose to this devastating event and discuss tailored strategies for prevention and repair.

## 2. Case Description

**Figure 1:**
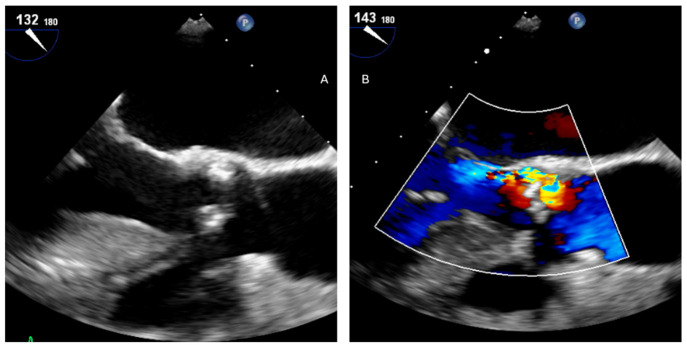
(**A**) Pre-operative TEE showing bicuspid valve with stenosis and (**B**) moderate regurgitation.

In October 2022, a 66-year-old woman with a bicuspid aortic valve and a low predicted operative risk, reflected by a EuroSCORE II of 2.01%, presented with symptomatic severe aortic stenosis associated with moderate aortic regurgitation (Figure 1). Preoperative transthoracic echocardiography demonstrated preserved left ventricular ejection fraction and concentric hypertrophy without additional structural abnormalities. Her body weight was 107 kg, and her surgical risk was considered low-to-intermediate. The patient underwent an elective surgical aortic valve replacement via a median sternotomy. After careful and complete annular decalcification, intraoperative sizing was performed strictly according to the manufacturer’s recommendations. Based on the annular assessment and obturator testing, a Perceval sutureless bioprosthesis (size XL) was selected. Deployment was technically straightforward without any post-implantation ballooning, resulting in a symmetrical expansion and stable anchoring.

**Figure 2:**
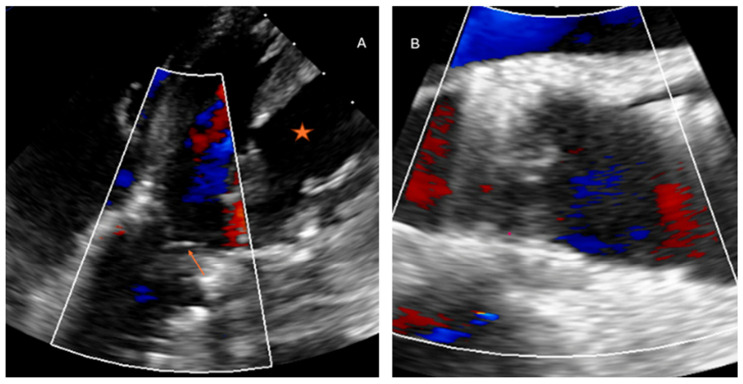
(**A**) Post implantation Perceval valve XL, no paravalvular leakage in diastole (arrow: cusps of Perceval closed), star: left ventricle; (**B**) good sitting position of Perceval valve.

Immediate intraoperative transesophageal echocardiography (TEE) demonstrated optimal prosthesis positioning, no paravalvular regurgitation, low transvalvular gradients (max 9 mmHg and mean 5 mmHg), and preserved biventricular function (Figure 2). There was no evidence of any septal disruption or perivalvular hematoma. The patient was extubated in the operating room and transferred to the intensive care unit in a stable hemodynamic condition.

**Figure 3:**
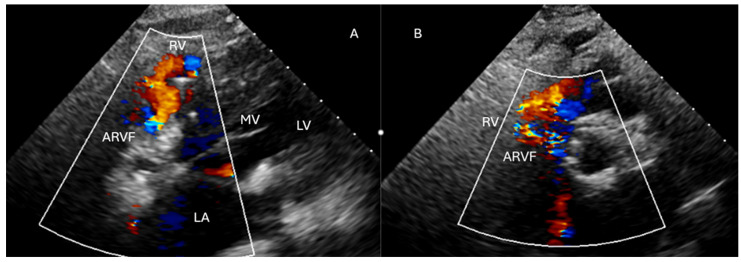
LA: left atrium, MV: mitral valve, LV: left ventricle, RV: right ventricle, ARVF: aorto-right ventricular fistula. (**A**) diastolic ARV shunts from the membranous septum tear to the right ventricle. The mitral valve is opened and the jet is in the opposite direction to the left chambers. (**B**) Another view showing the ARVF jet to the RV, from the side of the aorta.

Within the first 18 postoperative hours, she developed a complete atrioventricular block requiring temporary pacing. Shortly thereafter, she experienced acute hemodynamic deterioration characterized by hypotension, rising lactate levels, and escalating inotropic requirements, consistent with evolving cardiogenic shock. An urgent repeat TEE revealed a large communication between the aortic root and the right ventricle at the level of the membranous septum, producing a significant left-to-right shunt with a right ventricular volume overload (Figure 3). No prosthesis migration was observed, but the septal integrity was clearly compromised.

Given the rapid clinical decline, an emergent surgical re-exploration was undertaken. Intraoperative findings confirmed the tear of the membranous interventricular septum with a direct aorto-right ventricular fistulization. The Perceval prosthesis was carefully explanted to allow a full visualization of the rupture. The septal defect was closed using interrupted pledgeted sutures reinforced with a patch at the aorto-ventricular junction to restore structural continuity. A 27 mm Inspiris Resilia valve (Edwards Lifesciences, Irvine, CA, USA) was then implanted using a conventional sutured technique to ensure a controlled seating and minimize further septal stress. Due to a persistent high-degree atrioventricular block, a permanent epicardial pacemaker system was implanted during the same procedure. Post-repair hemodynamics remained unstable, necessitating the initiation of peripheral veno-arterial extracorporeal membrane oxygenation (VA-ECMO) via femoral cannulation for temporary circulatory support. Myocardial recovery allowed a successful ECMO weaning after three days. The postoperative course was complicated by critical illness neuropathy and intensive care unit–acquired weakness, but no recurrent shunt or prosthetic dysfunction was detected on serial echocardiography. The patient was discharged from the ICU on postoperative day 15 and transferred to a rehabilitation facility after a total hospital stay of five weeks. At the one- and two-year follow-up, she remains clinically stable and asymptomatic, with a normal prosthetic valve function, no residual intracardiac shunt, and satisfactory ventricular performance.

## 3. Discussion

Focusing specifically on a membranous septum rupture, this complication of ARVF remains exceedingly rare after aortic valve interventions and is far less documented than conduction disturbances. In transfemoral TAVR cohorts, its incidence has been estimated at 0.37% in a single-center series from 2012 to 2020 [3] and 1.5% in a 2015 multicenter registry of 400 Sapien (Edwards Lifesciences, Irvine, CA, USA) and CoreValve (Medtronic Inc., Minnesota, USA) implants [4]. Although the precise mechanism is not fully elucidated, it likely involves the chronic pressure exerted by an oversized or malpositioned prosthesis on the adjacent membranous septum, compounded by the potential septal penetration from stiff guidewires.

Case reports in the TAVR literature underscore this pathophysiology. An 87-year-old man developed a septal defect two years after a Sapien implantation, attributed retrospectively to valve oversizing [5]; similarly, a 70-year-old experienced both a septal rupture and an aortic root perforation after Sapien 3 deployment with post-TAVR oversizing again implicated [6]. Another CoreValve case in a 76-year-old highlighted a postoperative septal tear that was managed conservatively [7]. Such complications are virtually unreported following a Perceval valve implantation, underscoring their exceptional rarity within the sutureless AVR domain.

Beyond TAVR, two reports describe iatrogenic subaortic septal defects following a repeat conventional AVR: one in a patient undergoing sequential Mitroflow (Sorin Group USA Inc., Arvada, CO, USA) and then Epic valve (abbott, Chicago, IL, USA) replacements, and another after a combined mitral and aortic root surgery with Epic valves and a Dacron graft [8]. To our knowledge, only a single prior Perceval-related instance, a peri-membranous defect presenting two months postoperatively, has been documented [9]. Likewise, a lone report of an aorto-right ventricular fistula after surgical Perceval implantation exists, in which an annular rupture necessitated a percutaneous plug closure [10]. Against this backdrop, our case represents one of fewer than five published reports of a septal rupture or fistula complicating Perceval use, highlighting the need to scrutinize its unique risk factors.

Several predictive factors for conduction disturbances, and by extension septal trauma, have emerged with the Perceval prosthesis: valve oversizing, a shallow infra-annular septal length relative to the implantation depth, heavy annular calcification, and prior valve surgery [11,12,13,14,15]. Retrospective analyses demonstrate that avoiding the oversizing not only improves the hemodynamics and reduces the central regurgitation but also halves the new pacemaker rates (from 11% to 6.1%) [16,17,18,19]. Moreover, a short membranous septum significantly increases the risk of high-degree AV block, suggesting that an excessive septal compression may predispose both to a conduction injury and a mechanical rupture.

Preventive strategies have therefore focused on a meticulous sizing, using only the white obturator of the dedicated Perceval sizer to confirm an annular fit with slight friction, eschewing balloon dilation and optimizing the prosthesis depth to spare the membranous septum [20,21]. In one series, the deliberate leftward displacement of the septum after Perceval deployment in 11 patients yielded only mild conduction delays and no septal defects [22]. These tailored techniques underscore the delicate balance between a secure anchoring and the septal preservation.

The radial force exerted by the self-expanding Perceval valves plays a central role in achieving prosthesis stability and sealing within the aortic root. However, this same force may, under certain conditions, contribute to adverse structural outcomes. In vitro and computational studies led by Cabrera et al. [23] and Finotello et al. [24], have demonstrated that the radial force increases proportionally with oversizing, with forces exceeding 16 N associated with transmural arterial stress and potential vessel wall damage. Although Perceval valves generate lower radial force compared to balloon-expandable devices [20,25], oversizing beyond 22.6% has been linked to increased rates of degeneration [20], and in rare cases, complications such as a right aorto-ventricular fistula: this risk is particularly pronounced in patients with bicuspid aortic valves, whose aortic roots are known to exhibit congenital structural vulnerabilities [26].

The chronology of the fistula presentation further implies the radial force as a progressive, rather than an immediate, pathological factor. Multiple case reports, including those by Francisco et al. [10] and Hagiwara et al. [27], have documented fistulae developing between 3 days and one month post-implantation, suggesting an erosive mechanism rather than an intraoperative injury [28]. Unlike acute defects from ballooning or surgical trauma, these delayed lesions align more closely with a chronic pressure necrosis or the remodeling of the aortic wall in response to sustained mechanical loading. Simulation data support this interpretation, indicating that even in human arteries with relatively high circumferential stress thresholds (~290 kPa), the focal ‘von Mises stress’ may approach 210 kPa under oversizing conditions [24], which over time may exceed the adaptive capacity of the vessel wall. Beyond the nominal radial force, the biological impact of a self-expandable prosthesis depends on the stress transmitted to the surrounding tissue. Cabrera et al. demonstrated that increasing the oversizing progressively increases the arterial wall stress, with the von Mises stress highlighting areas of focal mechanical overload. This supports the concept that excessive oversizing may shift the prosthesis–tissue interaction from a stable anchoring toward tissue remodeling, injury, or perforation. This is particularly relevant in calcified or fibrotic roots, where the compliance is already compromised [20,26].

In the specific setting of a bicuspid aortic stenosis, both TAVI and Perceval share a biomechanical vulnerability: the implantation of a radially expanding prosthesis within an elliptical, asymmetric and often heavily calcified annulo-root complex. In TAVI, the persistence of the native bicuspid leaflets and a calcified raphe may impair the coaxial deployment and complete frame expansion, predisposing to an eccentric expansion, a paravalvular leak, a residual gradient, an annular/root injury, a coronary obstruction, embolic events and conduction disturbances. In contrast, the Perceval implantation benefits from the surgical valve excision and annular decalcification, but remains dependent on an accurate sizing, symmetric seating and annular circularization; type 0 bicuspid anatomy, a non-uniform commissural height and an unfavorable coronary-ostial orientation are recognized pitfalls. Thus, in a bicuspid anatomy, the radial force should be considered a double-edged mechanism: while necessary for anchoring and sealing, an excessive oversizing or asymmetric expansion may transform fixation into malexpansion, a leak, a conduction injury or structural tissue damage [29,30,31,32].

This is supported by the recent TAVI literature describing higher procedural complexity in BAV, especially with raphe calcification, tapered roots, PVL, PPI and annular complications, and by the sutureless-valve literature emphasizing oversizing, BAV morphology and root geometry as key determinants of the outcome [29,32].

Procedural adjuncts may further influence the biomechanical environment and exacerbate the effects of the radial force. The use of traction sutures at the level of the aortic commissures, especially when not removed after deployment, as in the modified technique described by Oldenburg [33,34], has been associated with an annular deformation and an altered stress distribution. Although this technique is not recommended by the manufacturer [35], it continues to be used at the surgeon’s discretion [34,36]. Vasanthan et al. emphasized the potential for unretrieved guiding sutures to cause deformation at the annular level, with possible consequences for the valve seating and force dynamics [37]. These alterations may amplify the localized stress and contribute to a delayed structural compromise. Considering these findings, further biomechanical research, ideally using cadaveric or ovine models focused on the Perceval valve in the aortic position, is warranted to better define the threshold forces and anatomical conditions that precipitate fistula formation.

In terms of management, the percutaneous closure of an aorto-right ventricular fistula has emerged as a viable, less invasive alternative in carefully selected cases, particularly when the patients are hemodynamically stable, the defect is focal and small (<5–8 mm), and there is no need for a concomitant valve intervention. Multiple case reports have demonstrated the successful deployment of Amplatzer vascular plugs or occluder devices under fluoroscopic and echocardiographic guidance, with relatively short procedural times, minimal blood loss, and same-day mobilization. Such an approach is especially appealing for elderly or high-operative-risk patients, as it avoids a sternotomy, a cardiopulmonary bypass, and the inflammatory sequelae of major surgery. However, in scenarios of an acute presentation, with a rapid cardiogenic shock, large-bore fistulas, or an associated prosthesis malposition, the surgical repair remains the gold standard. Open exploration permits a direct inspection of the rupture site, the complete removal of any damaged or malpositioned prosthesis, and a precise patch reconstruction of the membranous septum using bovine pericardium or synthetic materials. It also allows for the immediate re-implantation of a properly sized valve and the simultaneous management of conduction disturbances through epicardial pacing leads. In our patient, the combination of a sizeable septal tear, a perilous shunt volume, and the need for device explantation and pacemaker placement necessitated an urgent surgical revision.

## 4. Conclusions

Rupture of the membranous septum with an aorto-right ventricular fistula is an exceptionally rare but potentially life-threatening complication following Perceval valve implantation. Its occurrence underscores the critical importance of a meticulous valve sizing, specifically in a bicuspid valve anatomy and at a precise implantation depth to minimize the septal trauma. Management must be individualized: in the stable patients with small, delayed defects and a high surgical risk, the percutaneous closure offers a safe and effective alternative, whereas the acute, large-bore ruptures require prompt surgical repair. Moving forward, the systematic identification of anatomical risk factors and the ongoing refinement of sutureless AVR techniques will be essential to prevent both conduction disturbances and structural septal injuries.

## Data Availability

The original contributions presented in this study are included in the article. Further inquiries can be directed to the corresponding author.
